# A Higher Dosage of Oral Alendronate Will Increase the Subsequent Cancer Risk of Osteoporosis Patients in Taiwan: A Population-Based Cohort Study

**DOI:** 10.1371/journal.pone.0053032

**Published:** 2012-12-31

**Authors:** Wen-Yuan Lee, Li-Min Sun, Ming-Chia Lin, Ji-An Liang, Shih-Ni Chang, Fung-Chang Sung, Chih-Hsin Muo, Chia-Hung Kao

**Affiliations:** 1 China Medical University Hospital Taipei Branch, Taipei, Taiwan; 2 Graduate Institute of Clinical Medicine Science and School of Medicine, College of Medicine, China Medical University, Taichung, Taiwan; 3 Department of Radiation Oncology, Zuoying Armed Forces General Hospital, Kaohsiung, Taiwan; 4 Department of Nuclear Medicine, E-DA Hospital, Kaohsiung, Taiwan; 5 Department of Radiation Oncology, China Medical University Hospital, Taichung, Taiwan; 6 Management Office for Health Data, China Medical University Hospital, Taichung, Taiwan; 7 Ph.D. Program for Cancer Biology and Drug Discovery, China Medical University, Taichung, Taiwan; 8 Institute of Biomedical Sciences, Academia Sinica, Taipei, Taiwan; 9 Institute of Environmental Health, College of Public Health, China Medical University, Taichung, Taiwan; 10 Department of Nuclear Medicine and PET Center, China Medical University Hospital, Taichung, Taiwan; Georgia Health Sciences University, United States of America

## Abstract

**Background:**

Controversy still exists regarding whether alendronate (ALN) use increases the risk of esophageal cancer or breast cancer.

**Methods:**

This paper explores the possible association between the use of oral ALN in osteoporosis patients and subsequent cancer risk using the National Health Insurance (NHI) system database of Taiwan with a Cox proportional-hazard regression analysis. The exposure cohort contained 5,624 osteoporosis patients used ALN and randomly frequency-matched by age and gender of 3 osteoporosis patients without any kind of anti-osteoporosis drugs in the same period.

**Results:**

For a dose ≥1.0 g/year, the risk of developing overall cancer was significantly higher (hazard ratio: 1.69, 95% confidence ratio: 1.39–2.04) than in osteoporosis patients without any anti-osteoporosis drugs. The risks for developing liver, lung, and prostate cancers and lymphoma were also significantly higher than in the control group.

**Conclusions:**

This population-based retrospective cohort study did not find a relationship between ALN use and either esophageal or breast cancer, but unexpectedly discovered that use of ALN with dose ≥1.0 g/year significantly increased risks of overall cancer incidence, as well as liver, lung, and prostate cancers and lymphoma. Further large population-based unbiased studies to enforce our findings are required before any confirmatory conclusion can be made.

## Introduction

Alendronate (ALN) is the most common form of bisphosphonate used for the prevention and treatment of osteoporosis [1], [2]. Gastrointestinal toxicities are commonly seen in oral ALN users, and esophagitis is a well-known adverse effect of ALN use [3], [4]. Gastroesophageal reflux disease is an established risk factor for adenocarcinoma of the esophagus through the Barrett pathway [5]–[7]. Controversy still exists regarding whether ALN use increases the risk of esophageal cancer. Some studies suggested a possible increase in the risk of esophageal cancer [8], [9], but others have not found a relationship[10]–[12]. Conversely, several studies have reported that bisphosphonate use may be associated with a decreased risk of breast cancer [13]–[17], though observational studies may yield misleading results, and experts urge caution in interpreting results [18].

Oral ALN is widely used globally; therefore, a small magnitude of hazard could have important clinical implications, and it may attract public attention as well. A population-based large study may help clarify this controversy. We were interested in exploring this question and conducted a study using the database from the National Health Insurance (NHI) system of Taiwan.

## Materials and Methods

### Data Source

The present study used the reimbursement data of the universal NHI system in Taiwan, which registers all medical claims and has provided affordable healthcare for all residents in Taiwan since 1996. At the end of 2007, more than 99% of the population was enrolled in this insurance program, which contracted with 97% of clinics and hospitals. For administrative use and research, the National Health Research Institute (NHRI), Department of Health, established several randomly selected claim databases representative of the whole population. Sets of information available for the database cover all medical services received by each enrollee from 1996 to 2009, as well as characteristics of the patients, hospitals, and physicians. In this study, we used the insurance claims data of 1 million patients randomly selected from all enrollees in Taiwan in 1996–2000. We were able to use a scrambled identification number for each patient to link files, including the registry of medication prescribed, inpatient orders, and ambulatory care. Details of the database have been described previously [19]. Diagnoses were coded with the International Codes of Disease 9^th^ Edition Clinical Modification (ICD-9-CM). We confirm that all data was de-identified and analyzed anonymously. In addition, this study was also approved by the Ethics Review Board at China Medical University (CMU-REC-101-012).

### Study Sample

The study patients were identified in the database with newly diagnosed osteoporosis (ICD-9-CM 733.0) and underwent ALN treatment between 1998 and 2009 (n = 6,040). The date of the first ALN prescription was used as the index date. We excluded patients treated with other anti-osteoporosis drugs (n = 71) or with a cancer history predating the index date (n = 345). We finally extracted 5,624 patients to be study participants, defined as the ALN cohort.

For each of the remaining 5,624 patients taking ALN, we randomly selected three osteoporosis patients from the same period without any anti-osteoporosis drug treatment, and used the same exclusion criteria and frequency-matched with the case cohort for age and gender to establish the control group (non-ALN cohort) with totally 16,294 subjects. Moreover, we divided the case cohort into three groups according to the intake dosage of ALN: <1.0 g/year, 1.0–2.9 g/year, and ≥3.0 g/year.

### Study End Point

We linked the study patients to the registry of the Catastrophic Illness Patient Database (CIPD) to identify newly diagnosed cases of cancer using the unique patient identification number. The diagnosis of cancer in the National Health Insurance Research Database (NHIRD) requires histological confirmation and is reported in the CIPD. Each study patient was followed until a diagnosis of malignant cancer (ICD-9-CM 140–208) was made, until the patients were censored for loss to follow-up, death, or last withdrawal from NHI, or until December 31, 2009, the end of the follow-up (whichever came first).

### Statistical Analysis

Distributions of demographic characteristics including gender, age, occupation, and comorbidities were compared between patients with and without ALN treatment using chi-square tests. Poisson regression was used to calculate the incidence density and rate ratio of cancer by gender and age.

A multivariate Cox proportional hazard regression analysis was used to determine the effects of ALN use on the risk of cancer. The model was performed to calculate the hazard ratios (HRs) and 95% confidence interval (95% CI) of ALN use for gender, age, or specific cancer type. Cancer-free proportions were compared using the Kaplan-Meier method, and the three groups of ALN-intake dosage were estimated by a log-rank test. The Cox proportional hazard model assumption test was using scaled Schoenfeld residuals. There was no significant relationship between Schoenfeld residuals for cancer and follow-up time and it indicated that the assumption was fulfilled (p = 0.16).

All analyses were performed by SAS statistical software (version 9.1 for Windows; SAS Institute, Inc., Cary, NC, USA), and the significance level was set to 0.05.

## Results

We identified 5,624 osteoporosis patients treated with ALN between 1998 and 2009 as cases, and 16,294 osteoporosis patients without ALN as controls. Of the 5,624 cases, 84.1% of them were women (Table 1). No significant differences in gender (*P* = 0.45), age (*P* = 0.24), or comorbidities (*P* = 0.11 for hypertension, *P* = 0.95 for diabetes, *P* = 0.08 for hyperlipidemia) were found between ALN cases and controls.

**Table 1:** Baseline characteristics between ALN group and non-ALN group in 1998–2009.

Variables	Non-ALNN = 16,294		ALNN = 5,624		
	n	%	n	%	p-value
Median of follow-up year, (min, max)	3.04	(0.003–11.1)	2.92	(0.003–11.1)	
Sex					0.45
Women	13,770	84.5	4,729	84.1	
Men	2,524	15.5	895	15.9	
Age, years					0.24
<65	3,142	19.3	1,073	19.1	
65–74	5,850	35.9	1,972	35.1	
75–84	5,867	36.0	2,037	36.2	
≥85	1,435	8.8	542	9.6	
Occupation					0.03
Public^a^	1,832	11.2	668	11.9	
Labor	7,830	48.1	2,794	49.7	
Business	3,607	22.1	1,175	20.9	
Low income^b^	133	0.8	33	0.6	
Others	2,892	17.8	954	17.0	
Comorbidity					
Hypertension	11,929	73.2	4,179	74.3	0.11
Diabetes	4,411	27.1	1,520	27.0	0.95
Hyperlipidemia	7,175	44.0	2,400	42.7	0.08

a Government, education, and military.

b Insured income is lower than the level required for charging premium.

Chi-square test.

Table 2 shows the incidence densities and rate ratio of cancer by gender and age. Overall, there was no difference of cancer incidence rate between ALN cohort and non-ALN control group (12.2 per 1,000 person years vs. 11.1 per 1,000 person years, IRR = 1.10, 95% CI = 0.95−1.28). Similar results were observed when we separated women (11.2 per 1,000 person years vs. 10.4 per 1,000 person years, IRR = 1.08, 95% CI = 0.91−1.27) and men (19.8 per 1,000 person years vs. 16.6 per 1,000 person years, IRR = 1.19, 95% CI = 0.85−1.68). Male patients ≥75 years in the ALN cohort had highest cancer incidence (21.4 per 1,000 person years).

**Table 2:** Comparisons of incidence density of cancer between ALN group and non-ALN group by age and gender characteristics.

	Non-ALN			ALN				
Variables	Cases	Person-years	Rate†	Cases	Person-years	Rate†	IRR	(95% CI)
All								
<75 yrs	319	35,045	9.10	126	11,577	10.9	1.20	(0.97–1.47)
≥75 yrs	318	22,127	14.4	110	7,713	14.3	0.99	(0.80–1.23)
Overall	637	57,173	11.1	236	19,289	12.2	1.10	(0.95–1.28)
Women								
<75 yrs	265	31,664	8.37	105	10,420	10.1	1.20	(0.96–1.51)
≥75 yrs	258	18,645	13.8	85	6,547	13.0	0.94	(0.73–1.20)
Overall	523	50,309	10.4	190	16,967	11.2	1.08	(0.91–1.27)
Men								
<75 yrs	54	3,381	16.0	21	1,157	18.2	1.14	(0.69–1.88)
≥75 yrs	60	3,482	17.2	25	1,165	21.4	1.25	(0.78–1.99)
Overall	114	6,864	16.6	46	2,323	19.8	1.19	(0.85–1.68)

† per 1,000 person-year.

IRR, incidence rate ratio, compared to non-ALN group.

* p<0.05.

**Figure 1:**
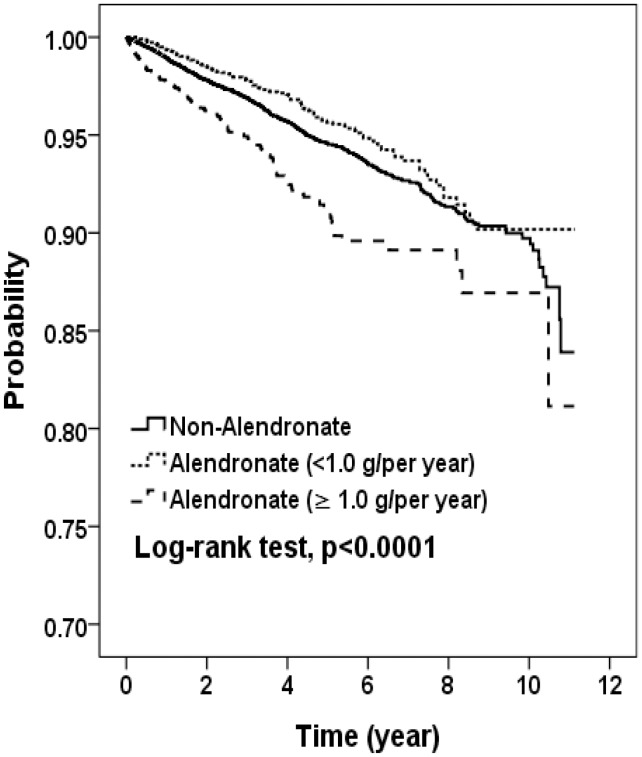
Kaplan-Meier model for estimating the cancer-free proportion of subjects.

ALN patients were grouped into three groups according to dosage: <1.0, 1.0–2.9 and ≥3.0 g/year. There were 3,073 patients treated low dosage, 1,358 patients treated median dosage and 1,193 patients treated with high dosage. The distribution of sex, age and comorbidity among four groups were no significant differences expect hyperlipidemia (p<0.05, data not shown). The risk of cancer associated with different dosage levels of ALNs shows that, compared to the non-ALN cohort, the adjusted HRs of cancer were increased with an increased dosage in the ALN cohort. The highest risk was in patients with a dosage ≥3.0 g/year (HR = 2.29, 95% CI = 1.76−2.99) (Table 3). We also observed that patients with a dosage <1.0 g/year had a lower risk of developing cancer than the non-ALN group (HR = 0.79, 95% CI = 0.64−0.96). The Kaplan-Meier analysis showed that the cancer-free rate was significantly less in the higher dosage ALN cohort than in the non-ALN cohort (log-rank *P*<0.0001; Fig. 1). Stratified HRs on gender showed similar trends in women and in men. For men in the ALN cohort, we then used a dosage <1.0 g/year as a reference group. The risk of cancer was highest with a dosage ≥3.0 g/year (HR = 3.08, 95% CI = 1.51−6.27).

**Table 3:** Hazard ratio of overall risk associated different dosage level of ALN use.

	N	HR	(95% CI)	HR	(95% CI)
All					
Compared group	637	1.00	(reference)		
ALN group (g/per year)					
<1.0	113	0.79	(0.64–0.96)*	1.00	(reference)
1.0–2.9	63	1.35	(1.04–1.75)*	1.72	(1.26–2.34)***
≥3.0	60	2.29	(1.76–2.99)***	2.92	(2.13–4.02)***
Women					
Compared group	523	1.00	(reference)		
ALN group (g/per year)					
<1.0	89	0.75	(0.60–0.94)*	1.00	(reference)
1.0–2.9	54	1.38	(1.07–1.82)*	1.82	(1.30–2.56)***
≥3.0	47	2.19	(1.62–2.95)***	2.91	(2.04–4.17)***
Men					
Compared group	114	1.00	(reference)		
ALN group (g/per year)					
<1.0	24	0.92	(0.59–1.43)	1.00	(reference)
1.0–2.9	9	1.21	(0.61–2.39)	1.33	(0.61–2.90)
≥3.0	13	2.281	(1.58–5.01)***	3.08	(1.51–6.27)**

Adjusted for sex, age, hypertension.

N: case numbers.

† per 1000 person-years.

* p<0.05, ** p<0.01, *** p<0.001.

The multivariate Cox proportional regression models specific to different types of cancer are presented in Table 4. Overall, compared with the non-ALN cohort, the ALN cohort was not associated with cancer risks in any individual site. However, stratified analysis on ALN dosage showed that, with a higher dosage (≥1.0 g/year), HRs increased significantly for all cancers (HR = 1.69, 95% CI = 1.39−2.04), liver cancer (HR = 1.94, 95% CI = 1.16−3.24), lung cancer (HR = 3.07, 95% CI = 1.97−4.76), prostate cancer (HR = 3.25, 95% CI = 1.43−7.36), and lymphoma (HR = 4.37, 95% CI = 1.49−12.8).

**Table 4:** Hazard ratios and 95% confidence interval of cancer associated with ALN in Cox’s regression analysis in different cancer.

					ALN dosage					
	Non-ALN	ALN			<1.0 g/per year			≥1.0 g/per year		
Variables	Cancercases	Cancer cases	HR	(95% CI)	Cancer cases	HR	(95% CI)	Cancer cases	HR	(95% CI)
All cancer	637	236	1.09	(0.94–1.26)†	113	0.79	(0.64–0.96)* †	123	1.69	(1.39–2.04)*** †
Oral cancer	15	2	0.40	(0.09–1.74)†	2	0.57	(0.13–2.48)†	0	–	
Esophagus cancer	8	3	1.10	(0.29–4.16)†	2	1.08	(0.23–5.08)†	1	1.15	(0.14–9.23)†
Stomach cancer	46	12	0.76	(0.40–1.44)†	8	0.77	(0.36–1.63)†	4	0.75	(0.27–2.09)†
Colorectal cancer	120	40	0.97	(0.68–1.39)†	18	0.66	(0.40–1.08)†	22	1.57	(1.00–2.48)†
Liver cancer	81	40	1.45	(0.99–2.12)†	22	1.20	(0.75–1.93)†	18	1.94	(1.16–3.24)* †
Lung cancer	76	38	1.47	(1.00–2.17)†	11	0.65	(0.34–1.22)†	27	3.07	(1.97–4.76)*** †
Breast cancer (only women)	48	17	1.06	(0.61–1.84)	9	0.85	(0.42–1.73)	8	1.46	(0.69–3.10)
Cervical cancer (only women)	31	5	0.47	(0.18–1.20)	2	0.28	(0.07–1.18)	3	0.83	(0.25–2.73)
Ovary cancer (only women)	5	1	0.58	(0.07–4.93)	1	0.90	(0.10–7.67)	0	–	
Endometrial cancer (only women)	5	3	1.79	(0.43–7.49)	2	1.76	(0.34–9.09)	1	1.85	(0.22–16.0)
Prostate cancer (only men)	21	13	1.83	(0.92–3.65)	5	1.08	(0.41–2.86)	8	3.25	(1.43–7.36)**
Bladder and kidney cancer	48	11	0.67	(0.35–1.29)†	5	0.46	(0.18–1.15)†	6	1.09	(0.46–2.54)†
Lymphoma	10	8	2.34	(0.93–5.94)†	3	1.32	(0.36–4.81)†	5	4.37	(1.49–12.8)** †
Other cancers	123	43	1.03	(0.73–1.45)†	23	0.82	(0.53–1.28)†	20	1.44	(0.90–2.31)†

ICD-9-CM: oral cancer, 140.xx, 141.xx, 143.xx-146.xx and 148.xx-149.xx; stomach cancer, 151.xx; colorectal cancer, 153.xx and 154.xx; liver cancer, 155.xx; lung cancer, 162.xx; breast cancer, 174.xx; cervical cancer, 180.xx; endometrial cancer, 182.xx; ovary cancer, 183.xx; prostate cancer, 185.xx; bladder and kidney cancer, 188.xx and 189.xx; Lymphoma, 202.xx.

Adjusted for age.

† Adjusted for age and sex.

* p<0.05,

** p<0.01,

*** p<0.001.

## Discussion

The results of adjusted analysis from this population-based cohort study indicate that exposure to oral ALN ≥1.0 g/year significantly increased the risk for overall cancer. For individual cancer risk, the use of oral ALN was not significantly associated with incident esophageal or breast cancer; conversely, we unexpectedly found significantly higher risks for liver, lung, and prostate cancers and lymphoma with higher ALN dose. These findings differ from those of prior literature reports.

Cancer has been the leading cause of death in Taiwan since 1982. The age-adjusted incidence rate has increased steadily, and it reached 270 new cases per 100,000 people in 2007 [20]. This trend is different from the United States Surveillance Epidemiology and End Results data, which showed that the overall cancer incidence rates in the U.S. decreased by 0.7% per year between 1999 and 2006 [21]. Because this problem continues to be a challenge for public health in Taiwan, it has come to the attention of the government, resulting in population-based investigations on cancer-preventive epidemiology. The NHI program provides comprehensive coverage, and the National NHIRD contains ambulatory service records, hospital service records, and prescription claims data. It allowed us to select appropriately matched patients representative of the underlying population. We recently used it to evaluate the risk of malignancy for patients with end-stage renal disease, and our article showed interesting findings [22]. The current study used a similar design and we attempted to determine whether use of oral ALN relates to the risk of cancer development.

A study from the University of Oxford indicated that the risk of esophageal cancer increased significantly by 2 times, with 10 or more prescriptions for oral bisphosphonates and with prescriptions over approximately a 5-year period [8]. Wysowski, from the Food and Drug Administration (FDA), suggested that the potential carcinogenic effect could be attributed to the esophagitis toxicity of oral bisphosphonates [9]. The current study does not reveal a relationship between oral ALN use and esophageal cancer, and agrees with reports from the United States and Europe [10]–[12]. However, the lack of a positive relationship in this study may also be because of the relatively small case number, which may not have enough power to detect differences between ALN and non-ALN patients.

Our results do not correspond with the findings of prior studies [13]–[17] because they showed a significantly lower risk for breast cancer in the oral ALN use group. The possible explanations could be as follows: 1) Female invasive breast cancer in Taiwan strikes at a relatively young median age (45–49 years) at diagnosis [23], and we can expect relatively fewer breast cancer patients diagnosed in older women with osteoporosis. This may weaken our statistical power to detect a significant difference. 2) Osteoporosis patients who take oral ALN tend to see doctors more frequently than those without medications, and have more opportunities for breast cancer screenings. We can expect more breast cancer cases to be detected in this group, which may weaken the possible protective role of ALN in breast cancer.

Table 3 revealed that the adjusted HRs of cancer were increased with an increased dosage in the ALN cohort, and it implied a dose-dependent association. We have no exact data regarding the severity of osteoporosis, but patients taking higher dose are supposed to have severer diseases based on an earlier study which found the dose-response relationships for ALN treatment in osteoporotic women [24]. Our data also showed that the adjusted overall cancer risk is significantly higher for the ALN group with a dose ≥1.0 g/year, and the main difference was contributed by liver, lung, and prostate cancers and lymphoma. This is a new finding, and limited related information can be found with an Internet search. Carcinogenicity studies in rats and mice at maximum tolerated doses showed no increased tumor incidence associated with ALN treatment [25]. In clinical use, the eHealthMe web site reported adverse drug effects from the FDA and user community, and recent data revealed that, among ALN users who reported side effects, 0.17% of them have lymphoma, 0.09% have prostate cancer, 0.06% have small-cell lung cancer, less than 1% (only 1 case) has stage IV non-small-cell lung cancer, and 0.33% have a liver disorder [26]. Although appropriate interpretation is difficult because of 1) no available comparison group, 2) uncertainty of data accuracy, and 3) lack of specification for certain toxicities, it can still offer some information regarding the safety of drug use. From the limited information available, we could not find any possible plausible mechanism to link the relationship between ALN use and risk of liver, lung, and prostate cancers or lymphoma. More frequent doctors’ visits by ALN users may partially explain the results. The Hepatitis B or C virus is well-known as a major risk factor in developing primary liver cancer. Taiwan was a high-prevalence area for hepatitis B and C, and hepatocellular carcinoma is common in Taiwan [27]. Hepatitis B or C carriers are advised to have periodic laboratory and abdominal ultrasonography exams. Patients with osteoporosis taking ALN are supposed to have more frequent clinical follow-up than patients with osteoporosis not taking any anti-osteoporosis drugs. Doctors may focus more on patients with comorbidity of hepatitis B or C and order some recommended exams, and more hepatocellular carcinoma could be diagnosed. The same explanation (more clinical check-ups, more cancer detection) could apply to lung and prostate cancers as well because both have easy methods of cancer screening. For lymphoma patients, the clinical symptoms/signs may alert patients/doctors, encouraging further evaluation, and more doctors’ visits offer a higher chance for early diagnosis of cancer.

Some may doubt whether a reverse-causality effect exists for the relationship between the use of ALN and cancer. To clarify the relationship, we incorporated a time lag between ALN exposure and cancer development into our model. Figure 1 demonstrates that the cancer-free proportions among patients treated with ALN ≥1.0 g/year, <1.0 g/year, and in the control group were significantly different over time. This indicates that cancer risks become increasingly different among the three groups over time, and the reverse-causality effect is less likely.

This study has the strengths in its large size, consequential period of follow-up, and use of NHIRD rather than self-reported drug use. However, certain limitations must still be addressed. First, detailed information such as smoking habits, alcohol consumption, body-mass index, socioeconomic status, and family history of cancer were unavailable from the NHIRD, all of which are major risk factors for multiple cancers and could plausibly be associated with osteoporosis and anti-osteoporosis medications. However, because NHIRD covers almost the whole population of Taiwan and the reimbursement policy is universal, these factors affecting the prescription of ALN would be unlikely. Second, the evidence derived from a cohort study is generally less reliable than that from randomized trials because a cohort study design is subject to numerous biases related to confounding adjustment. Despite our meticulous study design with adequate control of confounding factors, a key limitation is that bias could remain if unmeasured or unknown confounders exist. Third, diagnoses in NHI claims primarily serve the purpose of administrative billing and do not undergo verification for scientific purposes. We were unable to contact patients directly on their use of ALN because their identification numbers were anonymous. Prescriptions for these drugs before 1996 were not obtained in our analysis. This could cause underestimation of the cumulative dosage and may weaken the observed association. However, the data on the prescription of ALN and cancer diagnosis were highly reliable. Forth, several specific cancers analyzed in table 4 have very small case numbers. As such, the study may not have great power to detect differences between ALN and non-ALN patients.

In conclusion, this population-based retrospective cohort study did not find a relationship between ALN use and either esophageal or breast cancer. On the other hand, subgroup analysis found that higher dose of ALN was more likely to be related to the cancer risk, and use of ALN with dose ≥1.0 g/year significantly increased risks of overall cancer incidence, as well as liver, lung, and prostate cancers and lymphoma. Some undetermined underlying mechanisms remain to be explored. Further large population-based unbiased studies to enforce our findings are required before any confirmatory conclusion can be made.
